# Psychometric Properties and Gender‐Based Assessment of the Florence Emotional Eating Drive (FEED) in Brazilians

**DOI:** 10.1111/jhn.70229

**Published:** 2026-03-10

**Authors:** Carla Gonçalves Guareschi, Angela Nogueira Neves, Wanderson Roberto da Silva

**Affiliations:** ^1^ Postgraduate Program in Food, Nutrition and Food Engineering Sao Paulo State University (UNESP) Araraquara SP Brazil; ^2^ Postgraduate Program in International Security and Defense Army Physical Education School, Superior War College Rio de Janeiro RJ Brazil; ^3^ Centro Universitário de Patos de Minas (UNIPAM) Patos de Minas Minas Gerais Brazil

**Keywords:** drive, emotional eating, gender, psychometrics, questionnaire

## Abstract

**Background:**

Emotional eating, defined as eating influenced by emotional states, has been linked to the development of chronic diseases. Psychometric instruments, such as the Florence Emotional Eating Drive (FEED), are crucial for screening this behaviour, and their relevance must be validated in Brazil. This study aimed to evaluate the psychometric properties of the FEED in Brazilians and to examine gender‐based differences in emotional eating drive.

**Methods:**

This cross‐sectional study collected data online. The FEED originally comprises 23 items and three factors. Factorial, convergent, discriminant, and concurrent validity, as well as reliability, were analysed separately for each gender. Different factorial models of the FEED were tested, and refinement of a model previously applied in Brazil was required for both genders. FEED scores were computed, classified into categories, and compared between genders.

**Results:**

A total of 1274 individuals (54.6% men) aged ≥ 18 years participated. A 21‐item model with three factors (Brazilian refined version) demonstrated good psychometric properties for both men and women. A second‐order hierarchical model based on this structure was parsimonious and enabled the computation of a global emotional eating score. Significant gender differences emerged, with women presenting higher emotional eating scores. Most participants were classified as having no or low emotional urge to eat, with a higher prevalence among men.

**Conclusions:**

The FEED proved psychometrically robust for Brazilian women and men after item reduction. Gender‐based differences indicate greater emotional eating among women, reinforcing the importance of considering gender‐specific aspects in research and interventions addressing this construct.

## Summary

- The Florence Emotional Eating Drive (FEED) is a promising tool for assessing emotional eating among individuals aged ≥ 18 years in Brazil, with potential applications in research and population‐based screening of emotion‐related eating behaviours.
- Women presented higher emotional eating scores than men, highlighting the importance of gender‐sensitive counselling when assessing emotional eating patterns.
- The interpretation of FEED scores used in this study offers an exploratory approach to describing levels of emotional eating and may support food and nutrition education activities in both individual and collective contexts.

## 1 Introduction

Emotions are transient affective states that can trigger cognitive, physiological, and/or behavioural responses, varying in intensity and duration depending on the relevance of the stimulus [1, 2, 3]. These responses may affect the homeostatic regulation of the body and manifest through involuntary facial expressions, sweating, trembling, and other signs. In addition to influencing decision‐making processes and social interactions, emotions play a key role in eating behaviour, potentially modulating food choices, intake quantity, quality, and frequency of intake [4, 5]. Emotional eating has been described as the desire to eat in response to emotional states, rather than to physiological hunger cues [6]. In this context, emotional eating refers to changes in eating behaviour driven by negative, positive, or mixed emotions and has been widely investigated [7] in both non‐clinical and clinical populations [8].

Emotional eating may be linked to emotional dysregulation when it is recurrent and associated with difficulties in managing intense affective states. Emotional dysregulation, defined as the difficulty or inability to adequately regulate one's own emotions, impairs individuals' coping abilities in challenging situations, often leading to maladaptive behaviours across different contexts [4, 9, 10]. Evidence from clinical populations indicates that individuals with binge eating disorder and other eating‐related psychopathologies exhibit greater deficits in emotion regulation, which are strongly linked to patterns of emotional eating [9, 10]. Furthermore, emotional eating in clinical populations has been associated with depressive symptoms, body dissatisfaction, and the maintenance of disordered eating behaviours, reinforcing its role as a clinically relevant mechanism linking emotional dysregulation to adverse physical and mental health outcomes [9, 11]. Similar associations have also been observed outside clinical settings, as individuals who rely on food as a coping strategy for negative emotions frequently consume hyperpalatable foods, a pattern that may contribute to the development of noncommunicable chronic diseases, depressive symptoms, disordered eating, and body dissatisfaction [5, 12].

Within these non‐clinical contexts, emotional eating has been consistently associated with sex differences, with women showing higher susceptibility, particularly in response to negative emotions such as anxiety, sadness, and anger [13]. A study conducted in India with 102 participants found a 22% prevalence of emotional eating, which was more common among women reporting experiences of anxiety and anger [14]. Similarly, a study with 522 adults in Turkey revealed that women had the highest emotional eating rates, being nearly four times more likely to consume foods high in fat and sugar [15]. In Romania, a study with 300 participants showed that approximately 50% exhibited emotional eating behaviours, predominantly among women, who also tended to overestimate their body weight. A significant association was observed between body weight overestimation and emotional eating, suggesting that distorted body image may be linked to higher levels of emotionally driven food intake [11]. In Brazil, a study of 132 women aged between 18 and 60 years demonstrated that all phases of stress, such as alertness, exhaustion, and resistance, were associated with higher levels of emotional and external eating, leading to eating independent of the physical sensations of hunger and satiety and contributing to weight gain [13].

Psychometric instruments have been developed to track emotional eating, given the clinical and population‐level relevance of emotional eating. Among the most used instruments are the Emotional Eating Scale – EES [16], the *Dutch Eating Behaviour Questionnaire* – DEBQ [17], the *Emotional Appetite Questionnaire* – EMAQ [18], the *Positive‐Negative Emotional Eating Scale* – PNEES [19], and the *Florence Emotional Eating Drive* – FEED *Questionnaire* [20]. Each instrument offers distinct features for assessing emotional eating: some are broader in scope, such as the DEBQ, which also evaluates other types of eating behaviour; others, such as the EES, focus specifically on emotional triggers. While all these instruments are useful, some have been regarded as more informative due to their ability to more precisely capture emotional eating patterns, thereby supporting more targeted research and practice.

The FEED is considered a prominent tool for investigating emotional eating. Developed in Italy by Cassioli et al. [20], the instrument was based on the EES, using its set of negative emotions as conceptual starting points. However, rather than constituting a direct adaptation of the scale, the FEED introduces a novel measurement approach by jointly assessing the frequency with which specific emotions are experienced and the intensity of the eating urge elicited by those emotions. The final version focuses exclusively on negative emotional states and includes 23 items distributed across three factors (depression, anger, and anxiety) following an independent psychometric validation process. Each item is rated using two five‐point Likert‐type scales, which are subsequently combined into a composite score designed to better reflect the relevance of emotional eating.

The original validation study tested the FEED in a mixed sample comprising both clinical and non‐clinical participants. Specifically, the clinical sample included individuals with obesity, eating disorders, and type 2 diabetes, while a volunteer sample from the general population was also assessed. The instrument demonstrated excellent internal consistency, with Cronbach's alpha (α) values of 0.96 in the clinical sample and 0.89 in the non‐clinical sample. Convergent validity was supported by strong correlations between the total FEED score and the Binge Eating Scale – BES (*r* = 0.65, *p* < 0.001), as well as the EES (*r* = 0.78, *p* < 0.001). Additionally, the FEED showed significant associations with scores from the Eating Disorder Examination Questionnaire and the Symptom Checklist‐90‐Revised, which remained significant in multiple regression analyses, further reinforcing its convergent validity [20].

Despite robust psychometric evidence from the original Italian validation [20], the generalisability of the FEED beyond the Italian context had not yet been established at the time of this research. A Brazilian study [21] conducted a cross‐cultural adaptation of the FEED into Portuguese, reporting idiomatic, semantic, cultural, and conceptual equivalence. The adapted version was well understood by a target audience of Brazilian adults, and exploratory factor analysis (EFA) supported a three‐factor structure consistent with the original proposal; however, some items showed higher factor loadings on factors different from those originally specified. This indicates that, while the overall dimensional structure was maintained, the allocation of certain items across factors differed partially from the original version. Such discrepancies suggest potential cultural or linguistic influences and justify the need for further validation of the dimensional structure using confirmatory approaches [22].

Although instruments such as the DEBQ and EMAQ are also available in Brazil, the FEED stands out for its specific approach, as it assesses the presence of emotions before evaluating whether they trigger the urge to eat—an approach not commonly found in other tools. Therefore, the use of the FEED for tracking emotional eating in Brazil is promising because of its specificity and contemporary design. However, ensuring that the instrument provides accurate and reliable information about emotional eating is essential, which requires a psychometric validation study [23]. When an instrument demonstrates good validity and reliability, its results can support the development of effective strategies for the prevention and intervention of disorders involving dysfunctional eating behaviours that impact physical and psychosocial health. Accordingly, the present study aimed to evaluate the psychometric properties of the FEED, using a confirmatory approach, in a sample of Brazilians (men and women), and to examine gender‐based differences in emotional eating drive.

## 2 Methods

### 2.1 Study Design and Sample Size

This was a cross‐sectional study with a non‐probabilistic sample. The minimum sample size was determined based on recommendations from the literature [24], which suggests recruiting at least 15 participants per item of the instrument under investigation (FEED). Additionally, a 10% attrition rate, which is common in cross‐sectional studies, was considered. Based on these parameters, the minimum sample size was estimated at 384 participants. This target sample size was established for both women and men, as all analyses were conducted separately by gender.

### 2.2 Participants

Participants were individuals aged 18 years or older residing in Brazil. The inclusion criteria were being native Portuguese speakers and residing in the country. The exclusion criteria were pregnancy or the postpartum period (up to 9 months after childbirth) and currently undergoing treatment for chronic illnesses with a significant impact on eating behaviour. A screening questionnaire was administered prior to the full survey to confirm eligibility.

### 2.3 Procedures and Ethical Considerations

Data were collected online using Google Forms between November 2022 and August 2023. Recruitment was conducted via email, social media platforms (e.g., WhatsApp, Instagram, and Facebook), and in‐person through the researchers' personal networks. All participants provided electronic informed consent through a form presented at the beginning of the survey. They could withdraw at any time by discontinuing the questionnaire or requesting data deletion via email. At the end of the survey, participants were invited to share the study with their contacts, following a snowball sampling strategy [25]. Ethical approval was obtained from a Brazilian Research Ethics Committee (Brazilian Army Physical Training Centre, protocol number: 58840922.6.0000.9433), ensuring compliance with both national regulations and international ethical standards for research involving human participants. Artificial intelligence (ChatGPT‐5.2; OpenAI, San Francisco, CA, USA) was used exclusively for grammatical and language refinement under the authors' supervision.

### 2.4 Measures

A questionnaire was used to collect sociodemographic and health‐related data, including region of residence, marital status, race/skin colour, self‐identified gender, educational level, monthly household income, and self‐reported weight and height. Participants were also asked about clinical conditions (e.g., self‐reported eating disorder diagnosis), time spent on social media, engagement in dieting behaviours, and self‐perceived dietary quality.

The FEED was the primary instrument used in this study to evaluate its psychometric adequacy in the Brazilian samples. Developed by Cassioli et al. [20], the FEED assesses the frequency of emotions and the drive to eat in response to emotional states. The original factorial structure of the FEED comprises 23 items distributed across three factors: Depression (items 2, 5, 9, 10, 14, 15, 18, 21, and 23), Anger (items 1, 6, 8, 11, 16, 17, 19, and 20), and Anxiety (items 3, 4, 7, 12, 13, and 22). Each item corresponds to a specific emotion and is completed using two five‐point *Likert*‐type response scales: one for the frequency of the emotion (0 = never to 4 = always), and one for the urge to eat (0 = no desire to eat to 4 = overwhelming urge to eat). The authors proposed a combined 10‐point scoring system, ranging 0 (no urge to eat in the presence of the emotion) to 9 (maximum urge to eat due to the emotion).

In the present study, we used the original FEED, which was previously transculturally adapted into Brazilian Portuguese by Silva et al. [21]. In that Brazilian study, the authors identified the same three‐factor structure using EFA; however, the final model comprised 22 items, as item 6 (rebellious) did not demonstrate adequate factor loading and was therefore excluded. Furthermore, six items loaded on factors different from those proposed in the original model (marked with an asterisk), resulting in the following factor structure: Depression (items 1*, 2, 5, 7*, 9, 14, 15, 21, and 23), Anger (items 11, 12*, 16, 17, 18*, 19, and 20), and Anxiety (items 3, 4, 8*, 10*, 13, and 22).

To assess concurrent validity, we also applied the Brazilian version of the PNEES, which was likewise transculturally adapted into Brazilian Portuguese by Silva et al. [21]. The original PNEES [19] comprises 19 items measuring emotional eating in response to both negative (items 1, 2, 4, 6, 7, 8, 11, 12, 13, 15, 16, and 18) and positive (items 3, 5, 9, 10, 14, 17, and 19) emotional states. Responses are rated on a five‐point Likert‐type scale ranging from 0 (never) to 4 (very often). As the FEED specifically assesses eating drive in response to negative emotions, only the negative emotion subscale of the PNEES was used for concurrent validation. Although the PNEES includes both negative and positive emotional eating dimensions, these subconstructs, while correlated within the instrument's measurement model, represent distinct emotional valences and should be interpreted separately. This strategy was adopted to ensure conceptual correspondence between the instruments and to allow a theoretically grounded assessment of concurrent validity focused on negative emotional eating.

### 2.5 Data Analysis

All analyses were performed separately for women and men. Descriptive statistics were computed using IBM SPSS Statistics (version 22) to characterise the samples. Psychometric analyses of the FEED were conducted to examine its validity and reliability. These analyses were performed in R (version 4.1.3) using RStudio (version 2022.02.0 + 443) with the following packages: *lavaan* [26], *semTools* [27], and *psych* [28].

Initially, two previously proposed three‐factor models of the FEED were tested. First, we examined the original model comprising 23 items distributed across three factors as proposed in the validation study [20]. Subsequently, we examined the Brazilian model with 22 items, which also retained a three‐factor structure, but differed in the allocation of some items across factors, as suggested in the cross‐cultural adaptation study [21]. Overall, both models demonstrated acceptable factorial fit according to some indices; however, other parameters did not meet recommended criteria across samples. In particular, certain factor loadings and indicators of convergent validity were below acceptable thresholds in either men or women. Taken together, these findings indicated that, although the general factorial structure was supported, minor model refinement was necessary to achieve a more parsimonious and psychometrically robust measurement model applicable to both samples.

Psychometric evaluations were conducted in sequential steps. To assess psychometric sensitivity, we first examined item‐level descriptive statistics (mean, median, mode, standard deviation, skewness, and kurtosis). Next, we conducted confirmatory factor analysis (CFA) using the Weighted Least Squares Mean and Variance Adjusted (WLSMV) estimator, which is appropriate for instruments with ordinal indicators and latent constructs [22, 29]. Model fit was assessed using the following indices: Comparative Fit Index (CFI), Tucker–Lewis Index (TLI), Root Mean Square Error of Approximation (RMSEA) with 90% confidence interval, and Standardised Root Mean Square Residual (SRMR). Fit was considered good when CFI and TLI were ≥ 0.95, and RMSEA and SRMR were ≤ 0.08 [23, 30, 31]. Factor loadings (λ) < 0.50 were reviewed for potential exclusion [23, 32].

Convergent validity was evaluated using average variance extracted (AVE), with values ≥ 0.50 considered acceptable [23, 33, 34]. Discriminant validity was assessed by comparing the AVE of each factor with the squared correlation (*r*²) between factors, and was considered adequate when AVE > *r*². Concurrent validity was examined by correlating FEED and PNEES factors, with significant associations (*p* < 0.05) taken as evidence of validity. Internal consistency was assessed using the ordinal α coefficient [35] and omega (ω) coefficient [36], with values ≥ 0.70 indicating adequate reliability.

After identifying the best‐fitting model by gender, we calculated global emotional eating scores by averaging all FEED items. Additionally, factor‐specific scores were computed as the mean of their respective items. These scores were then compared between men and women using the Mann–Whitney *U* test, with a significance level of 5%. To facilitate interpretation, participants were classified into three categories of emotional eating (absence/low, moderate, and high) as follows: from 0 to 2.60 points = absence or low urge to eat in the presence of emotions; from 2.61 to 6.425 points = moderate urge to eat in the presence of emotions; and from 6.26 to 9.00 points = high urge to eat in the presence of emotions. This classification was applied to both global and factor scores, and gender differences in the distribution across categories were examined using 95% confidence intervals (95% CI). Importantly, this classification was developed for the present study, as no official cutoffs were proposed in the original publication [20] and no established clinical standards for emotional eating are currently available.

## 3 Results

A total of 1274 individuals (54.6% men) participated in the study. Three participants identified as another gender; due to the small number, they were not included in the analyses. The mean age was 33.4 ± 11.2 years for women and 31.3 ± 10.5 years for men. The mean body mass index (BMI) was 26.2 ± 5.8 kg/m² for women and 26.4 ± 4.6 kg/m² for men. Detailed characteristics of the sample are presented in Table 1.

**Table 1:** Participant characteristics.

Characteristic	Sample *n* (%)	
	Women	Men
Region of residence in Brazil		
North	28 (4.9)	168 (24.2)
Northeast	46 (8.0)	156 (22.4)
Midwest	49 (8.5)	32 (4.6)
Southeast	413 (71.5)	330 (47.5)
South	41 (7.1)	9 (1.3)
Marital Status		
Single	285 (49.3)	402 (57.9)
Married	188 (30.8)	190 (27.4)
Common‐law marriage	70 (12.1)	82 (18.8)
Separated/Divorced	42 (7.3)	18 (2.6)
Widowed	3 (0.5)	2 (0.3)
Skin colour/race		
White	381 (66.4)	299 (43.3)
Asian	12 (2.1)	9 (1.3)
Mixed‐race	134 (23.3)	291 (42.2)
Black	46 (8.0)	89 (12.9)
Indigenous	1 (0.2)	2 (0.3)
Educational level		
Completed elementary school	5 (0.9)	21 (3.0)
Completed high school	155 (27.1)	283 (40.9)
Completed undergraduate degree	163 (28.4)	233 (33.7)
Completed postgraduate degree	250 (43.6)	155 (22.4)
Monthly household income*		
≤ R$1,212 (USD 218.72)	75 (14.3)	111 (16.6)
R$1,213 to R$2,424 (USD 218.90–437.44)	97 (18.5)	122 (18.2)
R$2,425 to R$7,272 (USD 437.62–1,312.32)	204 (39.0)	254 (37.9)
R$7,273 to R$12,120 (USD 1,312.50–2,187.21)	89 (17.0)	139 (20.7)
≥ R$12,121 (USD 2,187.39)	58 (11.1)	44 (6.6)
How much time do you usually spend on social media for entertainment purposes on a typical day?		
< 1 h	68 (11.9)	175 (25.6)
1 to 3 h	313 (54.9)	375 (54.9)
3 to 6 h	146 (25.8)	109 (16.0)
> 6 h	42 (7.4)	24 (3.5)
Do you have any health condition that requires you to restrict certain foods from your diet?		
Yes	155 (27.1)	168 (24.5)
No	416 (72.9)	518 (75.5)
Have you ever received a medical diagnosis of an eating disorder?		
Yes	53 (9.3)	20 (3.0)
No	517 (90.7)	657 (97.0)
Have you ever gone on a diet to change your physical appearance?		
Never	100 (17.4)	203 (29.6)
Rarely	89 (15.5)	137 (20.0)
Sometimes	213 (37.1)	236 (34.5)
Frequently	120 (20.9)	77 (11.2)
Always	52 (9.1)	32 (4.7)
How would you rate the quality of your current diet?		
Poor	50 (8.7)	33 (4.8)
Fair	169 (29.2)	169 (24.5)
Good	194 (33.6)	276 (39.9)
Very good	133 (23.0)	159 (23.0)
Excellent	32 (5.5)	54 (7.8)
Anthropometric Nutritional Status^†^		
Underweight	8 (1.4)	4 (0.6)
Normal weight	280 (50.2)	273 (41.0)
Overweight	154 (27.6)	263 (39.5)
Obesity	116 (20.8)	126 (18.9)

*Note:* *In December 2025, 1 United States dollar (USD) was equivalent to approximately 5.54 Brazilian reais (BRL).

† Nutritional status based on body mass index (BMI) was classified according to the World Health Organisation (WHO) criteria.

Most participants reported residing in the Southeast region of Brazil, being single, identifying as white, spending one to 3 h per day on social media for entertainment purposes, and having a monthly income ranging from R$2,425 to R$7,272 (approximately USD 418 to 1,312). Most women reported having completed postgraduate education, whereas most men had completed high school. The majority of participants reported having engaged in dieting behaviours to modify their bodies at least occasionally and rated their dietary quality as good. Furthermore, most participants reported never receiving a medical diagnosis of an eating disorder and were classified as having an appropriate weight for their height (i.e., normal weight).

Table 2 presents the descriptive statistics used to assess the psychometric sensitivity of the FEED items. Skewness and kurtosis values were within acceptable ranges, with no severe violations of normality.

**Table 2:** Descriptive statistics and factor loadings of florence emotional eating drive (feed) items in brazilian samples.

Item	Women							Men						
	***M***	***Md***	***Mo***	*SD*	***Sk***	***Ku***	**λ**	***M***	***Md***	***Mo***	*SD*	***Sk***	***Ku***	**λ**
1	1.68	1	0	2.13	1.27	0.72	0.82	1.40	0	0	1.90	1.44	1.42	0.80
2	1.61	1	0	2.10	1.32	1.03	0.81	1.35	0	0	1.87	1.55	2.00	0.80
3	1.28	0	0	1.92	1.62	2.03		0.96	0	0	1.56	1.77	2.64	
4	3.44	4	0	2.54	0.28	−0.76	0.58	3.00	3	0	2.51	0.39	−0.83	0.65
5	1.31	0	0	2.09	1.81	2.67	0.72	1.12	0	0	1.83	1.86	3.06	0.75
6	0.84	0	0	1.50	2.20	5.43		0.83	0	0	1.58	2.30	5.40	
7	2.21	1	0	2.56	0.93	−0.27	0.84	1.87	1	0	2.29	1.17	0.55	0.77
8	4.26	4	0	3.16	−0.01	−1.38	0.84	3.81	4	0	3.01	0.19	−1.23	0.84
9	2.42	2	0	2.56	0.85	−0.29	0.86	2.08	2	0	2.23	0.96	0.11	0.83
10	2.97	2	0	2.58	0.51	−0.79	0.74	2.54	2	0	2.53	0.67	−0.65	0.78
11	2.12	2	0	2.40	0.98	0.09	0.86	1.93	1	0	2.27	1.13	0.49	0.89
12	0.64	0	0	1.32	2.59	7.60	0.61	0.63	0	0	1.29	2.61	7.76	0.66
13	0.99	3	0	2.71	0.45	−0.93	0.82	2.59	2	0	2.61	0.66	−0.64	0.80
14	2.24	2	0	2.40	0.94	−0.01	0.88	1.93	1	0	2.28	1.10	0.37	0.87
15	2.09	1	0	2.48	1.15	0.40	0.75	1.73	1	0	2.21	1.21	0.56	0.79
16	1.05	0	0	1.78	1.86	2.87	0.76	0.90	0	0	1.70	2.15	4.11	0.79
17	2.11	1	0	2.57	1.02	−0.04	0.72	1.85	0.50	0	2.41	1.21	0.54	0.76
18	1.44	0	0	1.94	1.41	1.47	0.79	1.26	0	0	1.91	1.67	2.35	0.80
19	2.06	1	0	2.27	0.88	−0.15	0.86	1.83	1	0	2.23	1.18	0.70	0.85
20	1.45	0	0	2.01	1.41	1.45	0.82	1.38	0	0	1.97	1.52	1.68	0.85
21	1.48	0	0	2.17	1.66	2.12	0.76	1.33	0	0	2.00	1.65	2.11	0.79
22	3.01	3	0	2.57	0.37	−0.97	0.71	2.59	2	0	2.57	0.65	−0.77	0.71
23	2.11	2	0	2.28	0.93	−0.09	0.87	1.73	1	0	1.99	1.14	0.82	0.84

*Note: M* = mean; *Md* = median; *Mo* = mode; SD = standard deviation; *Sk* = skewness; *Ku* = kurtosis; λ = item factor loading estimated from the best‐fitting measurement model for each study sample (see Table 3, Brazilian refined second‐order factor model).

Table 3 displays the psychometric properties of the tested FEED factor models. The original three‐factor model showed acceptable fit indices for CFI, TLI (> 0.90), and SRMR for both women and men, but inadequate RMSEA values. In addition, item 3 showed suboptimal factor loadings (0.40 for women and 0.38 for men), and the AVE for the anxiety factor was below acceptable levels (0.41 for women and 0.40 for men).

**Table 3:** Psychometric properties of different factor models of the florence emotional eating drive (feed) in brazilian samples.

Model (factors and items)	Sample	RMSEA (90% CI)	CFI	TLI	SRMR	λ	β	r	α	*ω*	AVE
Original	Women	0.10 (0.10–0.11)	0.94	0.93	0.07	0.40–0.86					
Depression (items 2, 5, 9, 10, 14, 15, 18, 21, 23)								0.92	0.93	0.92	0.61
Anger (items 1, 6, 8, 11, 16, 17, 19, 20)								1	0.91	0.90	0.59
Anxiety (items 3, 4, 7, 12, 13, 22)								0.97	0.78	0.77	0.41
Brazilian	Women	0.08 (0.08–0.09)	0.96	0.96	0.05	0.42–0.88					
Depression (items 1*, 2, 5, 7*, 9, 14, 15, 21, 23)								0.82	0.94	0.93	0.66
Anger (items 11, 12*, 16, 17, 18*, 19, 20)								0.91	0.91	0.89	0.61
Anxiety (items 3, 4, 8*, 10*, 13, 22)								0.85	0.83	0.84	0.49
Brazilian refined	Women	0.08 (0.08–0.09)	0.96	0.96	0.05	0.58–0.88					
Depression (items 1*, 2, 5, 7*, 9, 14, 15, 21, 23)								0.82	0.94	0.93	0.66
Anger (items 11, 12*, 16, 17, 18*, 19, 20)								0.92	0.91	0.89	0.61
Anxiety (items 4, 8*, 10*, 13, 22)								0.84	0.85	0.84	0.55
Brazilian refined unifactorial	Women	0.11 (0.11–0.12)	0.93	0.93	0.07	0.55–0.86					
Emotional Eating (all items together excluding 3 and 6)									0.96	0.95	0.56
Brazilian refined second‐order	Women	0.08 (0.08–0.09)	0.96	0.96	0.05	0.58–0.88					
Depression (items 1*, 2, 5, 7*, 9, 14, 15, 21, 23)							0.94		0.94	0.93	0.66
Anger (items 11, 12*, 16, 17, 18*, 19, 20)							0.86		0.91	0.89	0.61
Anxiety (items 4, 8*, 10*, 13, 22)							0.97		0.85	0.84	0.55
Emotional Eating (depression, anger, and anxiety factors)											
Original	Men	0.10 (0.09–0.10)	0.94	0.94	0.06	0.38–0.86					
Depression (items 2, 5, 9, 10, 14, 15, 18, 21, 23)								0.92	0.93	0.92	0.62
Anger (items 1, 6, 8, 11, 16, 17, 19, 20)								1	0.91	0.90	0.62
Anxiety (items 3, 4, 7, 12, 13, 22)								0.97	0.78	0.76	0.40
Brazilian	Men	0.08 (0.07–0.08)	0.96	0.96	0.05	0.40–0.89					
Depression (items 1*, 2, 5, 7*, 9, 14, 15, 21, 23)								0.85	0.94	0.92	0.65
Anger (items 11, 12*, 16, 17, 18*, 19, 20)								0.92	0.92	0.90	0.65
Anxiety (items 3, 4, 8*, 10*, 13, 22)								0.83	0.83	0.84	0.50
Brazilian refined	Men	0.08 (0.08–0.09)	0.96	0.96	0.05	0.65–0.89					
Depression (items 1*, 2, 5, 7*, 9, 14, 15, 21, 23)								0.85	0.94	0.92	0.65
Anger (items 11, 12*, 16, 17, 18*, 19, 20)								0.91	0.92	0.90	0.65
Anxiety (items 4, 8*, 10*, 13, 22)								0.82	0.86	0.85	0.58
Brazilian refined unifactorial	Men	0.11 (0.10–0.11)	0.93	0.93	0.06	0.62–0.85					
Emotional Eating (all items together excluding 3 and 6)									0.96	0.95	0.58
Brazilian refined second‐order	Men	0.08 (0.08–0.09)	0.96	0.95	0.05	0.65–0.89	0.98				
Depression (items 1*, 2, 5, 7*, 9, 14, 15, 21, 23)							0.87		0.94	0.93	0.65
Anger (items 11, 12*, 16, 17, 18*, 19, 20)							0.94		0.92	0.90	0.65
Anxiety (items 4, 8*, 10*, 13, 22)									0.86	0.85	0.58
Emotional Eating (depression, anger, and anxiety factors)											

*Note:* Items marked with an asterisk (*) indicate allocation to factors different from those proposed in the original model. The original model was proposed by Cassioli et al. (2022) and includes 23 items distributed across three factors. The Brazilian model was identified in a previous study by Silva et al. (2024) and comprises 22 items (excluding item 6) distributed across three factors. The Brazilian refined model was fitted in the present study with 21 items (excluding items 3 and 6) distributed across three factors. The Brazilian refined unifactorial model was tested in the present study with 21 items distributed across a single factor. The Brazilian refined second‐order model was tested in the present study with 21 items distributed across three first‐order factors and one second‐order factor.

*Abbreviations*: AVE = average variance extracted; CFI = Comparative Fit Index; *r* = correlations between first‐order factors (reported in the following order: depression vs. anger/depression vs. anxiety/anger vs. anxiety); RMSEA = Root Mean Square Error of Approximation; SRMR = Standardised Root Mean Square Residual; TLI = Tucker–Lewis Index; 90% CI = 90% confidence interval; λ = standardised factor loadings (range); α = ordinal alpha; β = second‐order factor parameter; ω = omega. Values for α, ω, and AVE are reported in the following order by factor: depression, anger, and anxiety.

Subsequently, a Brazilian model of the FEED, which was previously shown without item 6 [21], was tested. This model showed improved fit indices for both women and men; however, the AVE for women remained borderline (0.49), and item 3 continued to display loadings below 0.50 in both samples (0.42 for women and 0.40 for men). As a result, item 3 was removed from both samples, and a refined Brazilian model was tested. This 21‐item model (excluding items 3 and 6), comprising three factors, demonstrated adequate goodness‐of‐fit indices, with factor loadings ranging from 0.58 to 0.89, and satisfactory convergent validity across all factors.

Considering that Silva et al. [21] previously suggested that a unifactorial model could be plausible for the FEED, this structure was also tested after the exclusion of items 3 and 6. The unifactorial solution presented an acceptable, though comparatively inferior, goodness‐of‐fit when contrasted with the refined Brazilian three‐factor model. Given the empirical plausibility of both structures and the presence of consistently high correlations among the three first‐order factors in both samples (*r* = 0.82–0.92), a second‐order hierarchical model, based on the refined Brazilian model, was subsequently evaluated. This second‐order model demonstrated good overall fit, with all factor loadings exceeding 0.50 (see Table 3), and was therefore considered a parsimonious and theoretically coherent representation of the FEED factorial structure. Regarding reliability, all tested models showed good internal consistency, with both ordinal alpha and omega coefficients exceeding 0.70 across factors (see Table 3).

Table 4 shows the discriminant validity estimates for the FEED factors. Discriminant validity was observed only between the anger and anxiety factors in the male sample, as AVE value exceeded the *r* ^2^. Discriminant validity was not supported for the comparisons between depression and anger and depression and anxiety in either sample.

**Table 4:** Convergent, concurrent, and discriminant validity of the florence emotional eating drive (feed) based on the brazilian refined model.

	F1 (FEED)	F2 (FEED)	F3 (FEED)	F1 (PNEES)	F2 (PNEES)
F1 (FEED)	**0.66/0.65**	0.82/0.85	0.92/0.91	0.81/0.83	0.18/0.86
F2 (FEED)	0.67/0.72	**0.61/0.65**	0.84/0.80	0.66/0.64	0.31/0.66
F3 (FEED)	0.85/0.83	0.71/0.64	**0.55/0.58**	0.78/0.66	0.24/0.66
F1 (PNEES)	0.66/0.69	0.44/0.41	0.61/0.44	**0.62/0.62**	0.31/0.99
F2 (PNEES)	0.03/0.74	0.10/0.44	0.06/0.44	0.10/0.98	**0.67/0.64**

*Note:* Brazilian refined FEED: 21 items (excluding items 3 and 6) and three factors: depression (F1), anger (F2), anxiety (F3). PNEES = positive‐negative emotional EATING Scale, 19 items with two factors: negative emotional eating (F1) and positive emotional eating (F2). Diagonal values (in bold and a grey background) = average variance extracted (AVE) for each factor (convergent validity, ≥ 0.50). Values above the diagonal = correlation coefficients (r) between factors (concurrent validity). Values below the diagonal = squared correlations (r²) for discriminant validity (AVEi and AVEj ≥ r²ij). Values separated by a slash (/) refer to the women and men, respectively. All values were statistically significant (*p* < 0.05).

Table 4 also allows the evaluation of the concurrent validity of the FEED through its correlations with the PNEES. Prior to this analysis, the psychometric adequacy of the PNEES was confirmed in the present sample, as its two‐factor model showed satisfactory fit indices and high internal consistency for both women (CFI = 0.98; TLI = 0.98; RMSEA = 0.07; α = 0.93/0.95) and men (CFI = 0.98; TLI = 0.98; RMSEA = 0.06; α = 0.93/0.95). Furthermore, all AVE values for the PNEES exceeded 0.50, supporting convergent validity in both samples. Finally, all FEED factors were significantly (*p* < 0.05) correlated with both PNEES factors in women and men, providing evidence of concurrent validity. Stronger correlations were consistently observed between FEED factors and the negative emotional eating factor of the PNEES, whereas weaker correlations were found between FEED factors and the positive emotional eating factor of the PNEES among women.

Regarding FEED scores, women (w) had higher values than men (m) in the global score (w: median = 1.86, *IQR* = 2.57 *vs*. m: median = 1.62, *IQR* = 2.24), with a statistically significant difference (*U* = 221.752; *p* = 0.002). For the depression factor, women also scored higher (median = 1.44, *IQR* = 2.78) than men (median = 1.22, *IQR* = 2.33), showing a statistically significant difference (*U* = 171.302; *p* = 0.013). Similarly, for the anger factor, women had higher scores (median = 1.14, *IQR* = 2.14) than men (median = 1.00, *IQR* = 2.00; *U* = 214.325; *p* = 0.042). In the anxiety factor, women likewise scored higher (w: median = 3.20, *IQR* = 3.20 *vs*. m: median = 2.80, *IQR* = 3.20), with a statistically significant difference (*U* = 225,097; *p* < 0.001).

Table 5 summarises the classification of participants according to the global FEED score and its factors. Overall, most participants were classified as having no or low urge to eat in response to emotions, particularly among men. Gender differences were more evident in the depression and anxiety factors, with women showing a higher prevalence in the high category for depression, whereas distributions for the anger factor were largely similar between men and women. Notably, the anxiety factor showed a more balanced distribution across the absence/low and moderate categories, suggesting greater variability in emotional eating responses related to anxiety.

**Table 5:** Participant classification based on emotional eating scores according to the brazilian refined models of the florence emotional eating drive (feed).

Classification (urge to eat in the presence of emotions)	Sample			
	Women		Men	
	*n* (%)	95% CI	*n* (%)	95% CI
Global Score				
Absence or Low	416 (72.0)	68.3–75.6	560 (80.5)	77.6–83.3
Moderate	108 (18.7)	15.6–22.0	90 (12.9)	10.3–15.4
High	54 (9.3)	7.1–11.8	46 (6.6)	4.6–8.5
Depression Score				
Absence or Low	416 (72.1)	68.3–75.6	558 (80.2)	77.2–83.2
Moderate	88 (15.3)	12.5–18.2	88 (12.6)	10.2–15.4
High	74 (12.6)	9.9–15.4	50 (7.2)	5.2–9.1
Anger Score				
Absence or Low	365 (63.1)	59.3–67.1	479 (68.8)	65.5–72.4
Moderate	95 (16.4)	13.7–19.6	96 (13.8)	11.4–16.5
High	118 (20.5)	17.1–23.7	121 (17.4)	14.5–20.0
Anxiety Score				
Absence or Low	242 (41.9)	37.9–46.0	356 (51.1)	47.6–54.9
Moderate	231 (40.0)	35.8–44.3	248 (35.6)	32.0–39.2
High	105 (18.1)	15.1–21.5	92 (13.3)	10.9–15.8

*Note:* From 0 to 2.62 points: absence or low urge to eat in the presence of emotions; from 2.63 to 6.25 points: moderate urge to eat in the presence of emotions; and from 6.26 to 9.00 points: high urge to eat in the presence of emotions. 95% CI: 95% confidence interval.

## 4 Discussion

To the best of our knowledge, this is the first study to examine the psychometric properties of the Florence Emotional Eating Drive (FEED) in a Brazilian sample of women and men, in addition to assessing gender differences in emotional eating. Evaluating the measurement properties of a psychometric instrument is a crucial step in ensuring the validity and reliability of the data it produces. Accordingly, the present study evaluated the operationalization of the FEED using Brazilian data and subsequently examined the emotional eating behaviour of the participants. Validation procedures are not only methodologically important but also ethically necessary in research, as they help ensure that instruments yield accurate results and appropriately capture the constructs they are designed to measure.

In this study, two factorial models of the FEED were initially evaluated: the original 23‐item, three‐factor model [20] and a 22‐item, three‐factor model proposed in the Brazilian transcultural adaptation study [21]. Both models were tested via confirmatory factor analysis in Brazilian men and women, allowing for the evaluation of model fit across gender. However, neither model demonstrated adequate overall psychometric properties, indicating the need for model respecification, a common and recommended practice in psychometric research [23, 37].

When the original model [20] was evaluated, its fit indices did not reach recommended values, particularly the RMSEA; convergent validity was not achieved, and item 3 (“shaky”; translated into Portuguese as *“trêmulo”*) showed low factor loadings across both samples, indicating limitations in its psychometric performance. One possible explanation is that the formulation of this item may not sufficiently reflect emotional eating in response to negative affective states, particularly anxiety, the factor to which this item was originally assigned in the FEED. In Brazilian Portuguese, the term “trembling” can be interpreted broadly and does not necessarily imply an anxiety response related to suffering [1]. This lack of specificity may partly explain inconsistent interpretations by participants, thus weakening the association of the item with anxiety‐related emotional eating.

The Brazilian model, identified in the transcultural adaptation study [21], which excluded item 6 (“rebellious”; translated into Portuguese as *“rebelde”*), also failed to show satisfactory psychometric properties. In our analyses, item 6 emerged as problematic in both genders, suggesting that “rebellious” may not adequately capture the emotional antecedents of eating behaviour in the Brazilian context. Although the FEED original study [20] retained item 6 in the anger factor, the expression *“rebelde”* may convey a more behavioural or attitudinal meaning, rather than a direct affective state, weakening its conceptual alignment with this factor. Taken together, these findings support the exclusion of item 6, alongside item 3, from the refined model of the FEED for use in Brazil.

The refined Brazilian model, comprising three factors and 21 items (excluding items 3 and 6), demonstrated adequate fit indices, convergent validity for all factors, satisfactory factor loadings, and good internal consistency. These findings indicate that the terms “shaky” and “rebellious” may not be conceptually adequate to specific dimensions of emotional eating related to negative affect, such as anxiety‐ and anger‐related responses, aligning with diagnostic literature that characterises anxiety through fear and avoidance and anger through irritability and dysregulated emotional responses [38].

As suggested by Silva et al. [21], a unifactorial structure of the FEED was considered; however, in the present study, this model was tested using a refined set of 21 items, following item‐quality considerations. Although the unifactorial model showed acceptable fit, its fit indices were less favourable than those of the refined Brazilian three‐factor model, which provided a better fit to the emotional eating data in the current samples. Importantly, Silva et al. [21] employed exploratory factor analysis to assess the dimensionality of the FEED, whereas the present study empirically tested a predefined theoretical model using confirmatory factor analysis [23, 37]. Therefore, the discrepancies between the findings of the two studies are likely attributable to methodological differences.

Based on the observed pattern of results, a second‐order hierarchical model was also tested, given that the three‐factor model was specified oblique and exhibited high inter‐factor correlations. Such models are appropriate when first‐order factors reflect related dimensions of a broader underlying construct, allowing shared variance to be modelled at a higher level [23, 39, 40]. The hierarchical model yielded a good fit for both women and men, indicating that emotional eating can be conceptualised as a general construct encompassing correlated emotional dimensions, while still preserving meaningful distinctions among specific affective states [6, 41]. Although depression, anger, and anxiety are frequently associated with emotional eating [12], the literature indicates that emotional eating is not restricted to discrete emotional categories, as other affective states, such as stress, loneliness, and boredom, also play important roles in shaping eating behaviour [6, 16].

Correlations between FEED [20] and PNEES [19] factors showed a coherent pattern. As expected, all FEED factors were strongly correlated with the negative emotional eating factor of the PNEES, reflecting their close conceptual proximity and providing support for the concurrent validity of the instrument [6]. Conversely, weaker correlations were observed between FEED and the positive emotional eating factor of the PNEES, particularly among women. This pattern may reflect a greater responsiveness to eating in the presence of negative emotional states, a phenomenon previously documented in the literature [42, 43]. Nevertheless, because concurrent validity was examined using only one external instrument, these findings should be interpreted with caution. Future studies should include additional validated measures of emotional eating (e.g., DEBQ) to provide more comprehensive and robust evidence of concurrent validity.

Gender‐based comparisons revealed that women had higher FEED scores than men across all factors and in the global index. These findings are consistent with prior literature indicating greater female susceptibility to emotional eating, which can be understood as resulting from the interaction between biological and sociocultural determinants, including hormonal variations, lower levels of physical activity, and the intensification of sociocultural pressures associated with body weight and conformity to aesthetic standards [12, 44, 45]. Importantly, the difference observed in the anger factor was close to the threshold of statistical significance, suggesting that this result should be interpreted with caution and warrants further investigation in future studies. Although the strongest contrasts were found for the depression and anxiety factors, the overall consistency of the pattern across domains reinforces the robustness of the observed gender effect.

When the categorical classifications of emotional eating were examined, most participants were placed in the no/low category, with a higher proportion among men. In contrast, women were more frequently classified in the moderate category, reinforcing their greater susceptibility to emotional triggers for eating [46, 47]. No significant gender differences were observed in the global score within the high category. Men showed a higher prevalence in the no/low category for the depression and anxiety factors, whereas women presented a higher prevalence in the high category for the depression factor. These findings are consistent with studies showing that women are more strongly influenced by negative emotions, such as depression and anger, in their eating behaviour [48]. The more balanced distribution observed in the anxiety factor suggests that anxiety‐related eating behaviours are similarly represented across genders, unlike depression and anger factors.

This study has some limitations that should be acknowledged. First, there is the use of a non‐clinical sample composed exclusively of individuals from the general population. Although the original validation study of the FEED included both clinical and non‐clinical participants, the present study did not include a clinical subsample. Given that emotional eating tends to be more pronounced in clinical populations, this may limit the direct generalisation of the present findings to clinical contexts. This limitation may also partially affect conclusions related to the factorial structure, reinforcing the need for future studies to test and confirm the same measurement model in clinical samples.

Second, the classification of participants into emotional eating categories. This approach was adopted because no original cutoffs exist for this instrument. Therefore, this form of classification should be interpreted with caution, and further investigations are needed to establish clinically meaningful thresholds. Additionally, the sample, although large, was not representative of the Brazilian population, as it was predominantly composed of adults with higher education levels and from the Southeast region. This sociodemographic profile may limit the generalisability of the findings to groups with different cultural, socioeconomic, or educational backgrounds. Finally, self‐reported weight and height measurements were used to calculate BMI, which may have introduced measurement bias.

Despite these limitations, this study has several strengths. It is the first to evaluate the factor structure of the FEED in the Brazilian context using confirmatory factor analysis, providing a rigorous and theory‐driven assessment of its psychometric properties. This contribution is valuable to the literature on emotional eating because it underscores the importance of validating psychological instruments across cultural contexts. Another strength lies in the comprehensive methodological approach, which included testing multiple factorial models, evaluating different forms of validity and reliability, and applying a second‐order hierarchical model. Together, these strategies enhanced the robustness and credibility of the findings. Finally, the investigation of gender‐based differences in emotional eating added novel insights, extending the relevance of the FEED beyond psychometric evaluation and offering important implications for both research and screening protocols.

## 5 Conclusion

The present study evaluated the psychometric properties of the FEED in Brazilian women and men and examined gender‐based differences in emotional eating. A 21‐item, three‐factor model, which was also adequately represented by a second‐order hierarchical structure, showed the best fit to the Brazilian samples, supporting the measurement of emotional eating. Women consistently reported higher scores across FEED factors, while men were more frequently classified in the absence or low category of urge to eat in the presence of emotions, highlighting the importance of considering gender in the assessment of emotional eating. Although these findings provide evidence of adequate psychometric performance of the FEED in a Brazilian non‐clinical population, the original validation study also included a clinical sample, and the present study did not examine its properties in this context. Therefore, the same measurement model and factorial structure should be tested and confirmed in samples with clinically relevant eating‐related concerns, prior to its use in applied contexts focused on supporting behavioural change and promoting healthier eating‐related responses.

## Author Contributions

**Carla Gonçalves Guareschi:** Conceptualisation, Formal analysis, Methodology, Writing – original draft. **Angela Nogueira Neves:** Data curation, Formal analysis, Writing – review and editing. **Wanderson Roberto da Silva:** Conceptualisation, Methodology, Project administration, Supervision, Writing – review and editing.

## Ethics Statement

Ethical approval for the involvement of human subjects in this study was granted by the Research Ethics Committee of the Brazilian Army Physical Training Centre (protocol number: 58840922.6.0000.9433).

## Conflicts of Interest

The authors declare no conflicts of interest.

## Data Availability

Data will be made available on request.
